# A Moderated-Mediation Analysis of Organizational Justice and Leader-Member Exchange: Cross-Validation With Three Sub-samples

**DOI:** 10.3389/fpsyg.2021.616476

**Published:** 2021-06-25

**Authors:** Or Shkoler, Aharon Tziner, Cristinel Vasiliu, Claudiu-Nicolae Ghinea

**Affiliations:** ^1^HEC Montréal, Montréal, QC, Canada; ^2^Schools of Business Admin, Organizational Development and Counseling, Peres Academic Center, Rehovot, Israel; ^3^School of Business Administration, Netanya Academic College, Netanya, Israel; ^4^Faculty of Commerce and Tourism, Bucharest Academy of Economic Studies, Bucharest, Romania; ^5^Independent Researcher, Bucharest, Romania

**Keywords:** counterproductive work behavior, leader-member exchange, moderated-mediation, organizational citizenship behavior, organizational justice, work motivation

## Abstract

In an increasingly competitive work world, managers—whose links with subordinates, and their perceptions thereof, are critical components in that relationship—need to monitor employees' mindsets to facilitate their productivity. Our paper investigates organizational justice perceptions as an antecedent to two important outcomes: organizational citizenship behaviors and counterproductive work behaviors. The moderating effect of leader-member exchange and the mediating effect of work motivation were incorporated into a parsimonious moderated-mediation model designed to assist managers in achieving the stated objective. The model was tested on 3,293 Romanian workers, randomly divided into sub-samples of 1,098, 1,098, and 1,097 participants. Indicating high data consistency and credibility for the most part, in each sub-group, all the variables associated as predicted, with the notable exception of LMX. Implications, limitations, and suggestions for future research are discussed, with emphasis on the investigation's cultural context.

## Introduction

Traditionally, in the workplace, the relationship between employers and employees was marked by a top-down hierarchical arrangement whereby the association between the two parties was largely formal and authoritarian (Tziner and Rabenu, 2018). Workers were instructed to do a job for which they received due compensation and job security, and loyalty to the organization was a given. Today, it appears we are living and working in a new era where the dynamics between employers and their employees, especially in western, advanced societies, are rapidly changing.

This work world—primarily the product of advances in technological development, globalization, and increasing competition—has been outlined as VUCA (Volatile, Uncertain, Complex, and Ambiguous; see Bennett and Lemoine, 2014). To achieve a competitive advantage, organizations are increasingly hiring talent that is expert, skilled, and flexible. These individuals are highly knowledgeable, independent-minded, and not necessarily interested in staying in one place of work at any one time (Rabenu, 2021). Looking to the future, organizations are increasingly flat, teamwork is more widespread, and greater equanimity between employees and their managers is the order of the day (Tziner and Rabenu, 2018).

Under those circumstances, the need to draw out the best from workers is becoming an ever-greater challenge to management. To that end, we might ask what aspects of the work environment best enhance employees' motivations to be loyal, hardworking, and productive. Whether external to the workplace or pervading around workers on the job, the environment arouses feelings among the employees. The emotional baggage can be damaging, in which case adverse perceptions of the job experience are likely evident. Alternatively, the employees have an overall warm feeling about their work, which gives rise to positive responses to the job demands.

Management would want to have insight into the precursors of the positive perceptions likely to inspire their workers to be more amenable and productive at work. Of critical significance in the search for the links in that equation is the role of the leader-subordinate relationship. In sum, an appropriate research objective would be to derive a functional paradigm that highlights the links between employee perceptions and positive behaviors at work.

In our search for the answer to this salient objective, we adopted three well-known theories that underpin the dynamics of work interactions. The theories focus on (1) *social exchange* theory (SET; Blau, 1964), (2) *reciprocity* theory (Gouldner, 1960), and (3) *equity* theory (Adams, 1965) (see below) and precisely encounter mechanisms that influence people's affective states. Thus, the theories are pertinent to the work environment within which employees foster their emotions (e.g., Colquitt et al., 2009). Concerning the current investigation, we emphasize, in particular, the role of employees' of organizational justice perceptions (attitude) and work motivation (a dynamic state) derived from such mechanisms.

Thus, in the current research, we chose to tease out the relationships between a demarcated set of variables related to organizational justice perceptions, leader-member exchange (LMX), work motivation, and the outcomes of organizational citizenship behavior/workplace misbehavior, all of which been associated, on the one hand, with “negative” organizational events, such as: turnover (Bernerth and Walker, 2012) and burnout (Faragher et al., 2013) and, on the other hand, with enhanced productivity (Wang et al., 2010).

Underlying the theories is the notion that there is a mutual association between antecedents and outcomes, such that positive outcomes at work reinforce the antecedent behaviors (and vice versa). There is an underlying assumption that interactions in the workplace are much a give-and-take business, for better or worse (Gouldner, 1960; Blau, 1964; Adams, 1965). The individual worker will strive for a balance (equilibrium) between resources expended (such as time and effort) and outcomes (such as status, acknowledgment, and rewards). Imbalance (dissonance) would likely be rectified in destructive ways (Adams, 1965).

**Figure 1:**
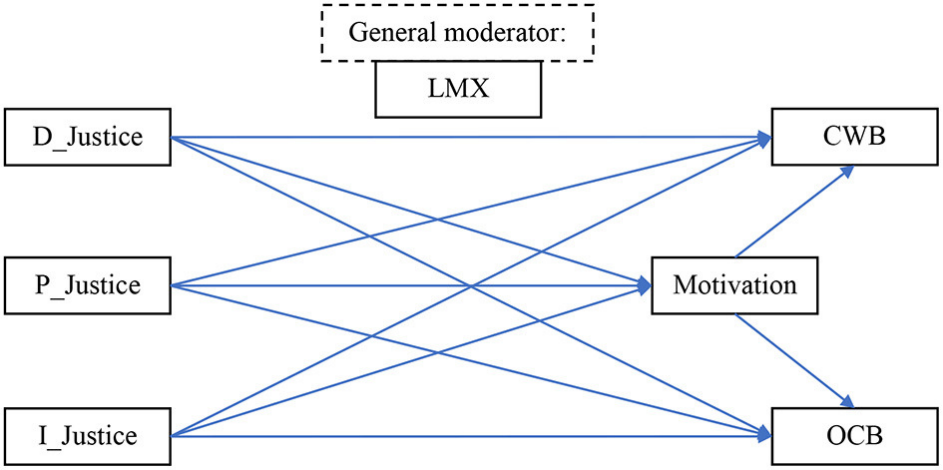
Model for the current research. D_Justice, distributive justice; P_Justice, procedural justice; I_Justice, interactional justice; LMX, leader–member exchange; CWB, counterproductive work behavior; OCB, organizational citizenship behavior.

Figure 1 outlines the proposed relationships between the variables. The ultimate objective of the model is to provide managers with a tool to measure (and predict) the potential productivity of their employees. In our model, perception of organizational justice serves as a relevant personal attribute to measure employees' attitudes to the work environment. LMX is seen as a potential mediator, and work motivation as a moderating variable on work productivity outcomes. Organizational citizenship behaviors (OCBs) and counterproductive work behaviors (CWBs) are considered likely work outcomes.

Specifically, we proposed to examine:

(1) The *association* between organizational justice perceptions (distributive, procedural, and interactional) and positive (i.e., OCB) and negative (i.e., CWB) outcomes;
(2) The role of work motivation as a *mediational* mechanism in our model; and
(3) The *moderating* effect of LMX on the overall model.

Notably, our investigation researches the association between the variables in the model employing the *moderated-mediation approach*, a seemingly under-used statistical approach. This is also called conditional indirect effects, and in this type of statistical analysis the effect of a predictor variable X on a(n) criterion/outcome variable Y through a mediator variable M differs depending on levels of a moderator variable Z. In other words, either the impact of X on M and/or the effect of M on Y depends on/conditioned by the level of Z (Muller et al., 2005; Preacher et al., 2007).

Thus, according to our model (Figure 1), the relationship between the precursor, personal levels of organizational justice (independent variable A), and possible outcomes (C) are affected by the levels of LMX (the mediator, B), which, in turn, moderates work motivation levels (D), that influence the degree to which the employees exercise OCB or indulge in CWB.

Notably, the associations among the variables in Figure 1 have been investigated (e.g., Eskew, 1993; Karriker and Williams, 2009; Al-A'wasa, 2018; Ugaddan and Park, 2019), but mostly in Western countries, including the USA, Australia, Canada, and the UK. To a much lesser extent, these relationships have been investigated in East-European or post-communist countries. Hence, Romania was chosen to be the focus of the study. Romania is an ex-communist working environment appeared to present a prime opportunity to test the universality of the relationships in our model. Of course, referring to Delery and Doty's (1996) contingency theory, we might expect that the operational exigencies operating in Romania at any one time to differ qualitatively from western modes of operation in the workplace—a theme to which we shall return.

## Central Attitudes and Critical Personal States

### Perceived Organizational Justice

Perceived organizational justice, a broad term to describe how employees view the manner in which they are treated in the workplace. Generally, a “high” level of perceived organizational justice would indicate, for instance, that employees are content with the level of information, resources, and feedback they receive or the degree of respect accorded them by superiors (e.g., Ambrose and Schminke, 2009).

Our research model broke the independent variable, organizational justice, into its three components: (a) distributive, (b) procedural, and (c) interactional justice (e.g., Colquitt et al., 2001). *Distributive justice* reflects perceptions regarding the fairness of outcomes, such as bonuses (see Adams, 1965), where notions of equality and equity play a role. *Procedural justice* reflects perceptions of the processes that lead to these organizational outcomes. These include ethics, accuracy, consistency, lack of bias, and representation of all concerned (Leventhal, 1980); managerial processes considered essential to maintaining institutional legitimacy. *Interactional justice* reflects the degree of fairness perceived in the way employers communicate or treat employees during the implementation of policies, procedures, processes, and outcomes. The underlying premise is that employees need to be treated with compassion, respect, dignity, and caring (e.g., Bies and Moag, 1986). We employed all three categories in our investigation.

When employees perceive that their relationship with their immediate manager/supervisor and their organization (as a whole) is satisfactory or balanced, they will be more disposed to mutually reciprocate by investing higher degrees of time, energy, creativity, and work-intensity behaviors (Pan et al., 2018). In other words, the employees are infused with high work motivation.

### Work Motivation

Tziner et al. (2012) indicated that work motivation is an inner mechanism that energizes individuals through thought and action to persevere until they achieve their goals. However, external forces also impinge on those processes. Pinder (2014, p. 11) extended that notion to incorporate an (additional) intrinsic energetic force that stirs the motivation beyond an individual's being. In the work context, these underlying energies initiate job-related behavior and “determine its form, direction, intensity, and duration” (Pinder, 1998, p. 11). In that vein, work motivation emanates from the *interaction* between the external organizational and societal environments and a person's characteristics (Latham and Pinder, 2005).

Often, the external forces are critical: a recession or pandemic can create stressful and uncontrollable pressures at work, to be blamed, perhaps, on the organization. However, in the daily run of things, as Fein and Klein (2011) commented, individual attributes constitute a significant source influencing value-laden perceptions and attitudes—and motivational levels subsequently—and the subjective assessment of the payoff of outcomes in the workplace.

In essence, we predict a flow of cause and effect: For instance, research has indicated that organizational justice correlates to high-quality LMX that, in turn, may lead to greater levels of mutual engagement, trust, and respect between managers and their employees (subordinates). Ultimately, the higher work motivation generated leads to enhanced attainment of work goals. Rewards follow, and they foster high organizational citizenship behaviors and low workplace misbehavior. In sum, the increased motivation drives the employee to higher levels of participation in the organizations' activities. Thus, in our present model, we highlighted perceptions of organizational justice as an individual antecedent to motivation.

### Organizational Justice and Work Motivation

Organizational justice, or employee perceptions of fairness, in the workplace appears to impact employees' drives to work. For example, workers who perceive that they are being treated fairly regarding bonus distribution or how managerial decisions are reached feel obliged to mutually reciprocate the fair treatment they received (Gouldner, 1960; Blau, 1964). Hence, a balance is maintained between employees' input at work (e.g., effort, expertise, knowledge) and what they receive in return (e.g., good/better working conditions, monetary compensation, job prestige, more challenging work) (e.g., Adams, 1965).

From the above, we arrived at the following hypothesis:

***H1: Organizational justice (distributive, procedural, interactional) positively*** ***associates with work motivation***.

### Organizational Citizenship Behavior

OCB consists of individual behaviors conducted by employees volunteering to contribute beyond their formal job duties to the organization, thus promoting its effective performance. The workers' contributions are discretionary, implicit, not overtly acknowledged by the organization's formal reward system (Organ et al., 2006). OCB is expressed in various forms, from dispositional tendencies (e.g., creativity and flexibility) to contextual factors (e.g., overtime and assisting colleagues) (e.g., Ahmad et al., 2020; Erum et al., 2020). These discretionary activities are greatly valued by management and represent an escalating contribution to the workforce, especially in today's increasingly dynamic and competitive organizational environment. Also, kindly refer to Podsakoff et al.'s (2009) meta-analysis in order to glimpse at the significance of this abounding phenomenon.

Among those contributions, we can recount that OCB enables the efficient allocation of limited resources by facilitating maintenance operations and freeing up resources for productivity (Organ et al., 2006). Furthermore, OCB allows workers and managers to carry out their jobs through more efficient and mindful scheduling, planning, and problem-solving (Podsakoff et al., 2009) while contributing to the quality of service (Lin et al., 2008). Organizations that nurture citizenship behavior are more attractive environments in which to work. They can hire the best employees and retain them (e.g., George and Bettenhausen, 1990). Because OCB is a *discretionary* indicator of loyalty and high motivation, it is highly pertinent that research seeks out those factors that augment or restrict OCB.

### Organizational Justice and OCB

Positive perceptions of organizational justice may invoke a greater work drive (i.e., motivation), an *attitudinal* outcome of such perceptions. However, as noted, the distributive, procedural, and interactional variants of OCB are also likely to be reciprocated by roughly-equal positive action (Gouldner, 1960; Blau, 1964). The workers' additional efforts “compensate” the perceived fair treatment (see also Ahmad et al., 2020). Thus, we hypothesize the following:

***H2: Organizational justice (distributive, procedural, interactional) positively*** ***associates with OCB***.

### Counterproductive Work Behavior and Workplace Misbehavior

In recent years, misbehavior at work has received increasing attention. On different sides of the same coin, a distinction has been made between (a) counterproductive work behaviors (CWBs) (Cohen-Charash and Mueller, 2007) and (b) workplace misbehaviors (WMBs). As implied, CWB is viewed by the organization as employees' deliberate actions operating against or in contrast to the organization's best interests (Gruys and Sackett, 2003, p. 30). The disreputable activities affect almost every aspect of the organization's functioning, including procedure, productivity, and, often, the workers themselves (e.g., Spector et al., 2006; Aubé et al., 2009). Concomitantly, CWB causes damage at all levels, psychological, sociological, and economic (Aubé et al., 2009; Bodankin and Tziner, 2009).

Consider, for example, the association between procedural *in*justice and CWB that might be mediated by the degree to which employees perceive a conflict between their work groups' norms and the organization's rules (“perceived normative conflict”) (Zoghbi Manrique de Lara and Verano Tacoronte, 2007). In such a case, the employees' perceptions lead them to a state of reluctance to comply with the rules of the organization (Cohen-Charash and Spector, 2001).

From the subjective stance of the offended worker, work misbehavior is manifested by a reduction of input into the job that inclines toward balancing the process of social exchange (Greenberg and Scott, 1996). Adverse reactions toward the organization run the whole gamut of attitudes and behaviors from lower motivational levels and distrust of higher authority to the point of criminal retaliation (e.g., Skarlicki and Folger, 1997; Spector et al., 2006).

### Organizational Justice and CWB

In that context, Chernyak-Hai and Tziner (2014) noted that the relationship between organizational justice and (CWB) manifests itself only when moderated by LMX. We suggest that the source of the employees' frustration with their supervisors might have been based on the employees' subjective feeling that their managers inappropriately rewarded them for the (high) investment of their personal resources. That perception lowers work motivation, and if the angst persists, the employees experience frustration. As indicated above, Chernyak-Hai and Tziner (2014) proposed that should employees encounter such imbalance and aversion, they would likely recoup the equilibrium through work misbehavior.

The effects that organizational justice perceptions have on behavior *at work* lead us to hypothesize that work motivation acts as a *mediator* in our model. That is to say that justice perceptions may affect workers' motivations to work—thus possibly eliciting enhanced positive or negative behaviors—independent of the *direct* effect of justice on the behavioral outcomes.

Based on the discussion above, we hypothesize further that:

***H3: Organizational justice (distributive, procedural, interactional) negatively*** ***associates with CWBs***.
***H4: (Work) Motivation mediates the relationships between organizational*** ***justice (distributive, procedural, interactional) and CWBs***.
***H5: Motivation mediates the relationships between organizational justice*** ***(distributive, procedural, interactional) and OCBs***.

### Conditional (Buffering) Effect—Leader-Member Exchange

Now we discuss the proposed *moderating* effect of LMX. The leader-member dyadic relationship, we recall, is by definition a two-way process. Thus, for each “member,” a unique response mode is called for by the “leader.” Employees, being individualistic, will also respond to their supervisors in their distinctive ways. As indicated, based on the theoretical models cited, the subordinates will be more or less obligated (or reluctant) to reciprocate depending on whether the LMX relationship is high or low.

Beyond reciprocity, the positive effects of high LMX are many. The fortunate employee enjoys higher respect and trust, feedback and support, rewards, and improved career opportunities (Clarke and Mahadi, 2017). These benefits, in turn, cause employees to exhibit further positive attitudes and behaviors, such as job engagement (Aggarwal et al., 2020), work commitment, and OCB (e.g., Chernyak-Hai and Tziner, 2014; Islam et al., 2020a,b; see Rockstuhl et al., 2012 for a comprehensive analysis). The employees also benefit from lowers levels of exhaustion, a primary source of burnout (e.g., Huang et al., 2010). For all these reasons, LMX is considered a critical constituent of the workplace social network (Cole et al., 2002).

We have expressed the importance we attach to the role of individual attributes. In the context of this investigation, it is expedient to emphasize the effects of individuals' dispositional differences on motivational levels and, particularly, on LMX, concerning which little research appears to have been conducted (e.g., Maslyn et al., 2017).

Furthermore, it appears that these relationships need to be studied in a broader range of cultural settings in order to establish the validity of the dyadic associations that appear to be consistent within a western setting (see Zagenczyk et al., 2015).

We return to the possibility that LMX serves as a moderator in our proposed model (see Figure 1) and reiterate the cause and effect nature of the LMX association. Thus, as intimated, the rewards (or otherwise) associated with LMX may profoundly influence employees' previously conceived attitudes to superiors at work. The more robust relationship with the managers is conducive to the internalization of (more) positive perceptions of justice. Thus, whatever opinions employees previously had of management may be moderated by the positive effect that the organizational justice has on their work motivation. As another example, high(er) LMX moderates the adverse effects deriving from justice perceptions that (in turn) gave rise to counterproductive work behavior. Based on this discussion, we hypothesize the following:

***H6: Leader-member exchange (LMX) moderates the associations in the model*** ***(i.e., as a general conditional factor)***.

### Hypotheses Summary

**H1**: Organizational justice (distributive, procedural, interactional) positively associates with work motivation.
**H2**: Organizational justice (distributive, procedural, interactional) positively associates with OCB.
**H3**: Organizational justice (distributive, procedural, interactional) negatively associates with CWBs.
**H4**: (Work) Motivation mediates the relationships between organizational justice (distributive, procedural, interactional) and CWBs.
**H5**: Motivation mediates the relationships between organizational justice (distributive, procedural, interactional) and OCBs.
**H6**: Leader-member exchange (LMX) moderates the associations in the model (i.e., as a general conditional factor).

## Methods

### Participants

In the current study, 3,293 Romanian subjects in the study, 39% males and 61% females between the ages of: 18–25 (53.5%), 26–35 (23.3%), 36–45 (12.5%), 46–55 (9.0%), 56–65 (1.7%), and 65+ (0.1%). In terms of education, respondents had either completed high-school education (31.2%), tertiary or post-secondary education (7.8%), they are holding/studying a Bachelor's degree (41.5%), they are holding/studying a Master's degree (19.3%), or they holding/studying a PhD (0.2%).

At work, most subjects held managerial positions (83.5%), including: (a) head of office or team (15.6%), (b) head of department (6.9%), or (c) director or executive manager (3.5%); the remaining participants of this managerial group (74.1%) were not at all responsible for the work of other people. Lastly, their tenure ranges between: (a) 0–5 years (66.2%), (b) 6–10 years (14.4%), (c) 11–15 years (7.6%), (d) 16–20 years (4.5%), (e) 21–25 years (2.9%), and (f) 25+ years (4.3%).

### Measures

#### Organizational Justice

Niehoff and Moorman's (1993) Justice Scale, comprising 20 items (Likert-types) between 1 (completely disagree) and 6 (completely agree), was employed as the measuring instrument. The measures reflected the three aspects of justice, as in the following examples: (1) *Distributive Justice*—“I consider my workload to be quite fair” (α = 0.83, *M* = 4.40, *SD* = 0.83); (2) *Procedural Justice*— “All job decisions are applied consistently across all affected employees” (α = 0.88, *M* = 4.43, *SD* = 0.97); and (3) *Interactional Justice*— “When decisions are made about my job, the general manager treats me with respect and dignity” (α = 0.89, *M* = 4.27, *SD* = 0.90).

*Work motivation*. We assessed this variable employing the Work Extrinsic and Intrinsic Motivation Scale (WEIMS; Tremblay et al., 2009). There are 18 items (Likert-type) range from 1 (does not correspond at all) to 6 (corresponds exactly). For example, “The reason for being involved in my job is the satisfaction I experience when I am successful at doing difficult tasks” (α = 0.91, *M* = 4.04, *SD* = 0.83).

#### Leader-Member Exchange

LMX was gauged by the Leader-Member Exchange Multi-Dimensional Measure (LMX-MDM attributed to Liden and Maslyn (1998). The measure includes 12 Likert-type items ranging from 1 (strongly disagree) to 6 (strongly agree). For example, “My supervisor would defend me to another in the organization if I made an honest mistake” (α = 0.85, *M* = 4.12, *SD* = 0.91).

#### Counterproductive Work Behavior

A scale by Bennett and Robinson (2000) (Interpersonal and Organizational Deviance Scale; IODS) was employed to measure CWB. The scale consists of 19 items (Likert-type) between 1 (never) and 6 (every day). For instance, “I deliberately worked slower than I could” (α = 0.95, *M* = 2.10, *SD* = 0.98).

#### Organizational Citizenship Behavior

OCB was gauged by a scale from Williams and Anderson (1991), namely, a 14-item scale (Likert-type) with response options between 1 (strongly disagree) and 6 (strongly agree). For example, “I help others who have been absent” (α = 0.83, *M* = 3.72, *SD* = 0.77).

### Procedure

We employed back-translation procedure suggested by Brislin's (1980). The items of the questionnaire were translated from English into Romanian. Care was taken to maximize semantic equivalence prior to the presentation of the questionnaire to end-participants. The translated questionnaires were administered by students (our research assistants) to respondents who formally consented that they wish to participate in our survey. The respondents were notified that the questionnaire was anonymous and confidential at all stages of its administration (acceding to the necessary legislation of the European Union concerning ethical standards).

## Results

### Common-Method Bias

Two methodologies were employed to test for the extent of possible common-method variance (CMV), accounting for variable intercorrelations in the results (see Podsakoff et al., 2003). The methods were: (a) Harman's single-factor method (all items are loaded into one common/marker factor) and (b) a common latent factor (CLF) method (all items are loaded into both their expected factors and one latent common method factor).

Based Harman's single-factor model, we notice that the results of the analysis accounted for only 25.49% of the explained variance (fit indices are suggested by, for example, Byrne, 2010; Islam et al., 2013; Shkoler and Tziner, 2017; Shkoler and Kimura, 2020): χ^2^(3, 070) = 9,433.57, *p* = 0.000, χ^2^/df = 3.07, CFI = 0.67, NFI = 0.66, GFI = 0.31, SRMR = 0.15, RMSEA (90% CI) = 0.24 (0.17–0.29), *p-close* = 0.000. Further, the CLF alternative model produced 23.17% of the explained variance: χ^2^(2, 991) = 7,115.34, *p* = 0.000, χ^2^/df = 2.38, CFI = 0.70, NFI = 0.69, GFI = 0.47, SRMR = 0.12, RMSEA (90% CI) = 0.14 (0.05–0.21), *p-close* = 0.001. Notably, these figures do not exclude the possibility of same-source bias (CMV). However, following Podsakoff et al. (2003), we note that if the explained variance accounted for by the first emerging factor is statured <50% (*R*^2^ < 0.50)—in conjunction with a poor model fit for each analysis—then the indication is that CMB is an improbable explanation of our findings.

Table 1 displays the zero-order intercorrelations in the research.

**Table 1:** Pearson correlation matrix.

	**1**	**2**	**3**	**4**	**5**	**6**	**7**
Distributive justice							
Procedural justice	0.84						
Interactional justice	0.87	0.88					
Motivation	0.53	0.56	0.54				
LMX	0.55	0.53	0.58	0.31			
CWB	−0.28	−0.27	−0.23	−0.15	−0.12		
OCB	0.34	0.33	0.35	0.27	0.33	−0.15	

*All the correlations are significant at p < 0.001. LMX, leader–member exchange; CWB, counterproductive work behavior; OCB, organizational citizenship behavior*.

**Figure 2:**
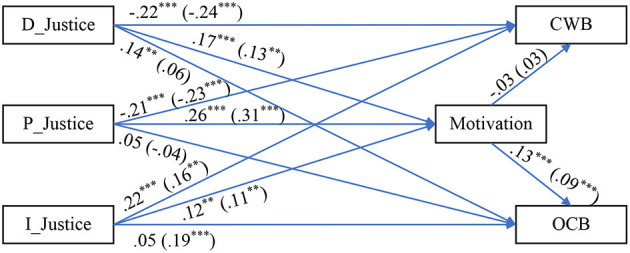
Path diagram with SEM results. Data outside parenthesis = Low LMX group. Data inside parenthesis = High LMX group. D_Justice, distributive justice; P_Justice, procedural justice; I_Justice, interactional justice; LMX, leader–member exchange; CWB, counterproductive work behavior; OCB, organizational citizenship behavior. **p < 0.01, ***p < 0.001.

To test the model (see Figure 1), we employed a SEM with multiple-group analysis using the AMOS software (v. 23). The model has fit in the absolute sense: $\chi_{\left(\text{df}\right)}^{2}$ = 22.35(11), *p* = 0.023, χ^2^/df = 2.04, SRMR = 0.03, GFI = 0.98, CFI = 0.99, NFI = 0.98, NNFI = 0.96, RMSEA (90% CI) = 0.06 (0.04–0.07), *p-close* = 0.479. Table 2 displays the findings from the path analysis made, while LMX is a moderator (*via* a Median-Split-Procedure: “*Low* LMX” = data *below or equal* to LMX's median, while “*High* LMX” = data *above* LMX's median), and also *Z*-tests to pinpoint where the differences in regression estimators, between the two LMX groups, are statistically significant. Also, Table 3 portrays the indirect effects analysis for the mediation effects. Figure 2 depicts the results on a path diagram.

**Table 2:** SEM path results with standardized regression coefficients and difference tests.

			***Low*** **LMX**		***High*** **LMX**		**Difference test**
**Path**			**β**	***Sig***.	**β**	***Sig***.	***Z*-score**
Distributive justice	→	Motivation	**0.18**	**0.001**	**0.14**	**0.002**	−0.34
Procedural justice	→	Motivation	**0.27**	**0.001**	**0.32**	**0.000**	1.28
Interactional justice	→	Motivation	**0.13**	**0.006**	**0.11**	**0.016**	0.14
Motivation	→	CWB	−0.04	0.310	0.02	0.207	1.62
Motivation	→	OCB	**0.12**	**0.000**	**0.10**	**0.000**	−0.84
Distributive justice	→	CWB	**−0.23**	**0.000**	**−0.25**	**0.000**	−0.69
Distributive justice	→	OCB	**0.15**	**0.002**	0.05	0.194	−0.97
Procedural justice	→	CWB	**−0.21**	**0.000**	**−0.22**	**0.000**	−0.70
Procedural justice	→	OCB	0.06	0.312	−0.03	0.478	−1.21
Interactional justice	→	CWB	**0.21**	**0.000**	**−0.17**	**0.004**	−2.50^**^
Interactional justice	→	OCB	0.06	0.324	**0.20**	**0.000**	2.18^*^

* *p < 0.05,*

** *p < 0.01. Bolded data are statistically significant. LMX, leader–member exchange; CWB, counterproductive work behavior; OCB, organizational citizenship behavior*.

**Table 3:** Mediation (indirect) effects analyses.

					***Low*** **LMX**			***High*** **LMX**		
**Paths**					**LL**	**UL**	***Sig***.	**LL**	**UL**	***Sig***.
Distributive justice	→	Motivation	→	OCB	0.01	0.05	0.004	0.01	0.04	0.011
Distributive justice	→	Motivation	→	CWB	−0.03	0.01	0.233	−0.01	0.03	0.160
Procedural justice	→	Motivation	→	OCB	0.03	0.07	0.000	0.02	0.06	0.003
Procedural justice	→	Motivation	→	CWB	−0.04	0.02	0.309	−0.02	0.05	0.207
Interactional justice	→	Motivation	→	OCB	0.02	0.05	0.000	0.01	0.04	0.003
Interactional justice	→	Motivation	→	CWB	−0.03	0.02	0.292	−0.01	0.03	0.144

*Analyses used bootstrapping (95% bias-corrected, 5,000 resamples). LL, lower limit of the CI; UL, upper limit of the CI; LMX, leader–member exchange; CWB, counterproductive work behavior; OCB, organizational citizenship behavior*.

As shown in Table 2, considering the between-groups comparison (Low LMX vs. High LMX), there are only two statistically significant differences in the correlational (bivariate) relationships between the variables. This finding designates that LMX is *not* a moderator.

Table 3 reveals that work motivation is a mediator, but *only* between the predictors: distributive, procedural, and interactional justice perceptions and the outcome: OCB. To the contrary, when CWB was the criterion, no mediation effect was found.

Table 4 summarizes the findings of the current research.

**Table 4:** Summary of results from hypotheses testing.

**Hypothesis/path**					***Low*-LMX**	***High*-LMX**
Distributive justice	→	Motivation			Supported	N.S.
Procedural justice	→	Motivation			N.S.	Supported
Interactional justice	→	Motivation			N.S.	Supported
Distributive justice	→	OCB			Supported	N.S.
Procedural justice	→	OCB			N.S.	N.S.
Interactional justice	→	OCB			N.S.	Supported
Distributive justice	→	CWB			N.S.	N.S.
Procedural justice	→	CWB			N.S.	Supported
Interactional justice	→	CWB			N.S.	N.S.
Distributive justice	→	Motivation	→	OCB	Supported	Supported
Procedural justice	→	Motivation	→	OCB	Supported	Supported
Interactional justice	→	Motivation	→	OCB	Supported	Supported
Distributive justice	→	Motivation	→	CWB	N.S.	N.S.
Procedural justice	→	Motivation	→	CWB	N.S.	N.S.
Interactional justice	→	Motivation	→	CWB	N.S.	N.S.
**LMX**	**=**	***Moderator***			**Supported**	

*N.S., not-supported; CWB, counterproductive work behavior; OCB, organizational citizenship behavior; LMX, leader–member exchange*.

### Further Analyses

As presented earlier in the paper, to test the research model (see Figure 1), a large sample was obtained, well above and beyond statistical requirements or rules of thumb. As such, we decided to divide this large sample into three randomly selected sub-samples to cross-validate the data and increase its credibility and accuracy.

**Figure 3:**
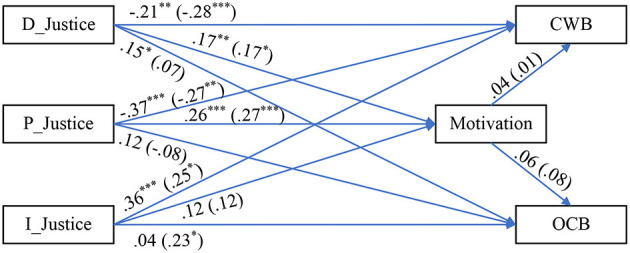
Path diagram with SEM results (sub-sample 1, *n*_1_ = 1,098). Data outside parenthesis = Low LMX group. Data inside parenthesis = High LMX group. D_Justice, distributive justice; P_Justice, procedural justice; I_Justice, interactional justice; LMX, leader–member exchange; CWB, counterproductive work behavior; OCB, organizational citizenship behavior. *p < 0.05, **p < 0.01, and ***p < 0.001.

**Figure 4:**
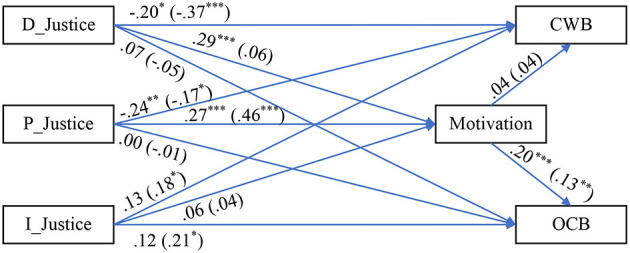
Path diagram with SEM results (sub-sample 2, *n*_2_ = 1,098). Data outside parenthesis = Low LMX group. Data inside parenthesis = High LMX group. D_Justice, distributive justice; P_Justice, procedural justice; I_Justice, interactional justice; LMX, leader–member exchange; CWB, counterproductive work behavior; OCB, organizational citizenship behavior. *p < 0.05, **p < 0.01, and ***p < 0.001.

**Figure 5:**
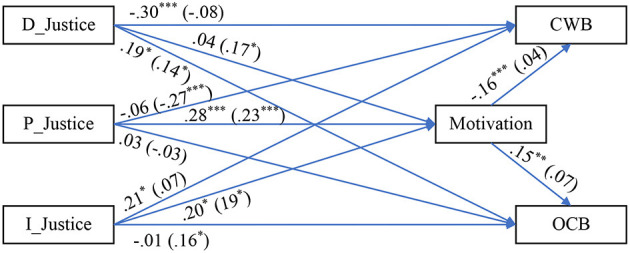
Path diagram with SEM results (sub-sample 3, *n*_3_ = 1,097). Data outside parenthesis = Low LMX group. Data inside parenthesis = High LMX group. D_Justice, distributive justice; P_Justice, procedural justice; I_Justice, interactional justice; LMX, leader–member exchange; CWB, counterproductive work behavior; OCB, organizational citizenship behavior. *p < 0.05, **p < 0.01, and ***p < 0.001.

Hence, three sub-samples, of almost equal size, were gleaned: (1) sub-sample 1 (*n*_1_ = 1,098), (2) sub-sample 2 (*n*_2_ = 1,098), and (3) sub-sample 3 (*n*_3_ = 1,097). We then proceeded to use these as the basis for replicating the analyses. The results are presented similarly to the Results section above, in Tables 5–8 and Figures 3–5. In other words, we repeated the same analyses and data presentation format following the Results section, once for each sub-sample.

**Table 5:** Means, standard deviations and reliability coefficients for each sub-sample.

	**Sub-sample 1**^****a****^			**Sub-sample 2**^****b****^			**Sub-sample 3**^****c****^			**Total sample**^****d****^		
**Variable**	***M***	***SD***	**α**	***M***	***SD***	**α**	***M***	***SD***	**α**	***M***	***SD***	**α**
Distributive justice	4.44	0.94	0.84	4.39	0.92	0.83	4.38	0.93	0.83	4.40	0.93	0.83
Procedural justice	4.44	0.99	0.89	4.42	0.95	0.88	4.42	0.97	0.87	4.43	0.97	0.88
Interactional justice	4.29	0.92	0.90	4.26	0.89	0.89	4.26	0.90	0.89	4.27	0.90	0.89
Motivation	4.05	0.86	0.91	4.00	0.82	0.90	4.09	0.81	0.91	4.04	0.83	0.91
LMX	4.13	0.91	0.84	4.11	0.89	0.84	4.11	0.93	0.86	4.12	0.91	0.85
CWB	2.07	0.97	0.95	2.15	0.98	0.95	2.08	0.97	0.95	2.10	0.98	0.95
OCB	3.74	0.79	0.84	3.72	0.74	0.82	3.70	0.76	0.82	3.72	0.77	0.83

a *n = 1,098.*

b *n = 1,098.*

c *n = 1,097.*

d *N = 3,293. LMX, leader–member exchange; CWB, counterproductive work behavior; OCB, organizational citizenship behavior*.

**Table 6:** Pearson correlation matrix.

	**1**	**2**	**3**	**4**	**5**	**6**
D_Just	–					
P_Just	0.84/0.85/0.84/0.84	–				
I_Just	0.87/0.87/0.86/0.87	0.88/0.89/0.88/0.88	–			
Mot	0.53/0.53/0.53/0.52	0.56/0.55/0.56/0.55	0.54/0.54/0.23/0.55	–		
LMX	0.55/0.56/0.50/0.57	0.53/0.54/0.50/0.55	0.58/0.57/0.57/0.60	0.31/0.34/0.25/0.33	–	
CWB	−0.28/−0.28/−0.31/−0.23	−0.27/−0.29/−0.30/−0.23	−0.23/−0.22/−0.26/−0.19	−0.15/−0.15/−0.15/−0.17	−0.12/−0.16/−0.12/–**0.07**	–
OCB	0.34/0.36/0.31/0.35	0.33/0.35/0.31/0.33	0.35/0.36/0.33/0.28	0.27/0.26/0.32/0.28	0.33/0.35/0.32/0.34	−0.15/−0.12/−0.14/−0.84

*The correlations are presented as follows: total sample (N = 3,293) **and then** sub-sample 1 (n_1_ = 1,098) **and then** sub-sample 2 (n_2_ = 1,098) **and then** sub-sample 3 (n_3_ = 1,097); i.e., total/1/2/3. All the correlations are significant at p < 0.001 (apart from a bolded correlation which is significant at p < 0.05). Data on the diagonal in bold and parentheses are the reliability coefficients (Cronbach's alphas). D_Justice, distributive justice; P_Justice, procedural justice; I_Justice, interactional justice; Mot, motivation; LMX, leader–member exchange; CWB, counterproductive work behavior; OCB, organizational citizenship behavior*.

**Table 7:** SEM path results with standardized regression coefficients for each sub-sample.

			**Sub-sample 1 (*****n***_****1****_ **=** **1,098)**				**Sub-sample 2 (*****n***_****2****_ **=** **1,098)**				**Sub-sample 3 (*****n***_****3****_ **=** **1,097)**			
			***Low*** **LMX**		***High*** **LMX**		***Low*** **LMX**		***High*** **LMX**		***Low*** **LMX**		***High*** **LMX**	
**Path**			**β_**1**_**	***Sig*._**1**_**	**β_**1**_**	***Sig*._**1**_**	**β_**2**_**	***Sig*._**2**_**	**β_**2**_**	***Sig*._**2**_**	**β_**3**_**	***Sig*._**3**_**	**β_**3**_**	***Sig*._**3**_**
D_Justice	→	Mot	**0.17**	**0.020**	**0.17**	**0.020**	**0.29**	**0.000**	0.06	0.447	0.04	0.544	**0.17**	**0.012**
P_Justice	→	Mot	**0.26**	**0.001**	**0.27**	**0.000**	**0.27**	**0.000**	**0.46**	**0.000**	**0.28**	**0.000**	**0.23**	**0.000**
I_Justice	→	Mot	0.12	0.128	0.12	0.166	0.06	0.376	0.04	0.682	**0.20**	**0.018**	**0.19**	**0.010**
Motivation	→	CWB	0.04	0.477	0.01	0.770	0.04	0.386	0.04	0.388	**−0.16**	**0.000**	0.04	0.371
Motivation	→	OCB	0.06	0.195	0.08	0.085	**0.20**	**0.000**	**0.13**	**0.007**	**0.15**	**0.002**	0.07	0.161
D_Justice	→	CWB	**−0.21**	**0.010**	**−0.28**	**0.000**	**−0.20**	**0.011**	**−0.37**	**0.000**	**−0.30**	**0.000**	−0.08	0.307
D_Justice	→	OCB	**0.15**	**0.048**	0.07	0.396	0.07	0.385	−0.05	0.589	**0.19**	**0.017**	**0.14**	**0.044**
P_Justice	→	CWB	**−0.37**	**0.000**	**−0.27**	**0.002**	**−0.24**	**0.004**	**−0.17**	**0.047**	−0.06	0.495	**−0.27**	**0.000**
P_Justice	→	OCB	0.12	0.200	−0.08	0.381	0.00	0.987	−0.01	0.941	0.03	0.734	−0.03	0.750
I_Justice	→	CWB	**0.36**	**0.000**	**0.25**	**0.011**	0.13	0.136	**0.18**	**0.045**	**0.21**	**0.024**	0.07	0.449
I_Justice	→	OCB	0.04	0.691	**0.23**	**0.019**	0.12	0.155	**0.21**	**0.038**	−0.01	0.943	**0.16**	**0.049**

*Bolded data are statistically significant. D_Justice, distributive justice; P_Justice, procedural justice; I_Justice, interactional justice; Mot, Motivation; LMX, leader–member exchange; CWB, counterproductive work behavior; OCB, organizational citizenship behavior*.

**Table 8:** Mediation (indirect) effects analyses for each sub-sample 1.

					***Low*** **LMX**			***High*** **LMX**		
**Paths**					**LL**	**UL**	***Sig***.	**LL**	**UL**	***Sig***.
*Sub-sample 1* (*n*_1_ = 1,098)										
Distributive justice	→	Motivation	→	OCB	0.02	0.07	0.009	0.01	0.04	0.007
Distributive justice	→	Motivation	→	CWB	−0.04	0.00	0.210	−0.00	0.06	0.177
Procedural justice	→	Motivation	→	OCB	0.01	0.05	0.000	0.02	0.10	0.011
Procedural justice	→	Motivation	→	CWB	−0.04	0.02	0.357	−0.01	0.04	0.253
Interactional justice	→	Motivation	→	OCB	0.00	0.03	0.000	0.01	0.05	0.013
Interactional justice	→	Motivation	→	CWB	−0.02	0.09	0.402	−0.02	0.01	0.166
*Sub-sample 2* (*n*_2_ = 1,098)										
Distributive justice	→	Motivation	→	OCB	0.00	0.05	0.006	0.00	0.05	0.015
Distributive justice	→	Motivation	→	CWB	−0.02	0.02	0.194	−0.00	0.04	0.152
Procedural justice	→	Motivation	→	OCB	0.02	0.08	0.000	0.03	0.08	0.008
Procedural justice	→	Motivation	→	CWB	−0.03	0.00	0.231	−0.04	0.01	0.199
Interactional justice	→	Motivation	→	OCB	0.01	0.08	0.000	0.00	0.06	0.014
Interactional justice	→	Motivation	→	CWB	−0.01	0.03	0.167	−0.00	0.02	0.145
*Sub-sample 3* (*n*_3_ = 1,097)										
Distributive justice	→	Motivation	→	OCB	0.01	0.09	0.011	0.01	0.07	0.013
Distributive justice	→	Motivation	→	CWB	−0.03	0.01	0.255	−0.02	0.06	0.140
Procedural justice	→	Motivation	→	OCB	0.03	0.09	0.000	0.01	0.07	0.005
Procedural justice	→	Motivation	→	CWB	−0.06	0.02	0.338	−0.03	0.05	0.285
Interactional justice	→	Motivation	→	OCB	0.02	0.05	0.000	0.02	0.10	0.021
Interactional justice	→	Motivation	→	CWB	−0.03	0.03	0.352	−0.01	0.03	0.173

*Analyses used bootstrapping (95% bias-corrected, 5,000 resamples). LL, lower limit of the CI; UL, upper limit of the CI; LMX, leader–member exchange; CWB, counterproductive work behavior; OCB, organizational citizenship behavior*.

Table 9 summarizes the findings of the current research, for each sub-sample.

**Table 9:** Summary of results from hypotheses testing (sub-sample 2, *n*_2_ = 1,098).

					**Sub-sample 1**		**Sub-sample 2**		**Sub-sample 3**	
**Hypothesis/path**					***L*-LMX**	***H*-LMX**	***L*-LMX**	***H*-LMX**	***L*-LMX**	***H*-LMX**
Distributive justice	→	Motivation			Sup.	Sup.	Sup.	N.S.	N.S.	Sup.
Procedural justice	→	Motivation			Sup.	Sup.	Sup.	Sup.	Sup.	Sup.
Interactional justice	→	Motivation			N.S.	N.S.	N.S.	N.S.	Sup.	Sup.
Distributive justice	→	OCB			Sup.	N.S.	N.S.	N.S.	Sup.	Sup.
Procedural justice	→	OCB			N.S.	N.S.	N.S.	N.S.	N.S.	N.S.
Interactional justice	→	OCB			N.S.	Sup.	N.S.	Sup.	N.S.	Sup.
Distributive justice	→	CWB			Sup.	Sup.	Sup.	Sup.	Sup.	N.S.
Procedural justice	→	CWB			Sup.	Sup.	Sup.	Sup.	N.S.	Sup.
Interactional justice	→	CWB			Sup.	Sup.	N.S.	Sup.	Sup.	N.S.
Distributive justice	→	Motivation	→	OCB	Sup.	Sup.	Sup.	Sup.	Sup.	Sup.
Procedural justice	→	Motivation	→	OCB	Sup.	Sup.	Sup.	Sup.	Sup.	Sup.
Interactional justice	→	Motivation	→	OCB	Sup.	Sup.	Sup.	Sup.	Sup.	Sup.
Distributive justice	→	Motivation	→	CWB	N.S.	N.S.	N.S.	N.S.	N.S.	N.S.
Procedural justice	→	Motivation	→	CWB	N.S.	N.S.	N.S.	N.S.	N.S.	N.S.
Interactional justice	→	Motivation	→	CWB	N.S.	N.S.	N.S.	N.S.	N.S.	N.S.
**LMX**	**=**	***Moderator***			**Sup**.		**Sup**.		**Sup**.	

*Sup., supported; N.S., not-supported; L-LMX, low LMX; H-LMX, high LMX; CWB, counterproductive work behavior; OCB, organizational citizenship behavior; LMX, leader–member exchange*.

In sum, the analyses revealed that the three sub-samples demonstrate similar, but not identical, relationships to the total sample. This finding further augments the credibility of the data, results, and implications.

## Discussion

The goal of the current paper was to shed light on: (1) the relationship between organizational justice perceptions (distributive, procedural, and interactional) and positive (i.e., OCB) and negative (i.e., CWB) outcomes; (2) the mediational effect(s) of work motivation in the model; and (3) the moderation effect(s) of LMX in the model (see Figure 1). To this end, we employed a large-scale study in an East-European country: Romania.

The results revealed that most of our hypotheses were corroborated: (H1, H2, and H3) organizational justice (distributive, procedural, interactional) negatively associates with CWB and positively with work motivation and OCB; (H4) work motivation did not mediate between organizational justice and CWB; (H5) work motivation mediated *only* two of these relationships (first, distributive justice-motivation-OCB. Second, or procedural justice-motivation-OCB); and (H6) the LMX level, as a moderator, appeared to be a conditional factor in model, albeit *only partially*.

### Implications and Future Suggestions

1. The overall results of the investigation replicate previously revealed associations between the variables in the model, albeit not totally, with the exception of the moderating effect of LMX. At the most basic level, we recommend that managements internalize the possible debilitating effects of their workers' negative perceptions of organizational justice in all its manifestations. Organizations are urged to create just and fair work environments that promote positive motivations and OCB while reducing counterproductive work behaviors—benefitting both the organizations and their employees.

Furthermore, we also recommend that management consistently monitors the motivation levels of their employees. As observed, work motivation acts as a *partial* mediator to OCB (i.e., justice → motivation → OCB). Management is thus encouraged to extend the opportunities to raise motivation at work and, consequently, increase OCB, among other positive outcomes in the workplace.

2. We note that in the final analysis, despite indications both theoretical and empirical, LMX did *not* moderate any of the relationships in the model as hypothesized (see Figure 1). That is to say that the exchanges between managers and their subordinates do not appear to act as a conditional factor. Several considerations might explain this outcome:

First, the result obtained in this investigation may simply correspond to Chernyak-Hai and Tziner's (2014) observation that the predicted organizational justice/counterproductive work behavior (CWB) relationship exists *only* when it is moderated by the *extent* of leader-member exchange. That is to say, that the composite (mean) measure of LMX in each of the three sub-groups was simply not sufficiently high to achieve the expected result.

It would also appear that the assumptions noted in the introductory discussion did not hold with this set of subjects. That is to say, the respondents of the survey did not necessarily view low-LMX as depletion of their resources. Nor did they view any negative perceptions entertained as a reaction to inappropriate rewards for the investment of their valuable personal resources.

Second (and likely related to the last comment), the above result was obtained in the specific Romanian cultural context, only two decades removed from its associations with Soviet culture. The possibility arises that the questionnaires employed in our investigation, and designed in the West, were not appropriate for the Romanian workers' mindset, even though they were semantically adapted to the Romanian language, as noted in the Method section. Furthermore, among the respondents, there may yet have been a lingering distrust of surveys of any kind that emerge from “higher authorities,” a residual hangover from the Soviet system.

Moreover, beyond the challenges of reliability represented by surveys associated with misbehaviors at work (see Limitations, below), it is quite conceivable that what the Romanian subjects responded to on paper did not adequately reflect their true feelings or work behaviors. This supposition would apply primarily to LMX and perceptions of fairness at work, whereby norms that apply in the western world do not necessarily apply to the Romanian society, only recently having emerged from a repressive ethos. Put bluntly: “However the supervisor acts toward me is a bluff.” This assumption is supported somewhat by Zagenczyk et al.'s (2015) observation that a “mismatch” between expectations from favorable LMX relationships and work outcomes can be a reality in the workplace. In their words: “Employees may have LMX perceptions which are *inconsistent* with the favorability of treatment that they receive” (Zagenczyk et al., 2015, p. 105).

Thus, while the model replicates previous findings in some respects, we cannot ascertain that the current results of this investigation *apropos* the LMX moderating effect are valid universally or that the surveys, in and of themselves, were reliable in the Romanian context.

Alternatively, we note the several references in our discussion to the effects of external and internal influences on employees' attributes, attitudes, and internal states. In contradistinction to external influences on the workplace, we chose to emphasize those individual characteristics that influence the build-up of positive and negative behaviors on the job. That the exchanges between employees (i.e., subordinates) and their managers did not appear to act as a conditional factor in our investigation could be explained by asserting that, specifically in the Romanian context, extraneous *external* factors impacted the respondents in a manner that militated against the effects of LMX on workers' behavior in their work environments.

Consider, for example, that there may be an unveiled cognitive process of attribution that should be explored in the future. Indeed, in contrast to the wary, conservative attitude described above, we could adduce that the (external) surrounding work ethos in Romania may be such that ex-Soviet Romanian employees would never even contemplate the thought that their immediate managers were unfair. Moreover, recalling the traditional, authoritarian approach to work and productivity in the open lines of our discussion, we could feasibly conceive that compliant Romanian workers are suspicious of attempts to intrude into their personal space. Thus, at work or when responding to questionnaires, the employees are reticent, despite the degree to which the experimenters complied with the ethical demands of the investigation.

Based on these kinds of presumptions, we recommend (1) adapting the surveys to the normative behaviors and attitudes that define the Romanian workplace and (2) replicating the study in various countries and cultural settings. These future studies would ultimately augment the external validity of the research (On the significance and value of replications, see Tziner, 2018).

Further, we recommend that future research focus on additional potential moderators and, specifically, on what might be labeled the classical internal indicators of individual differences or attributes that serve as predictors. These indicators include emotional intelligence and the Big Five personality factors (openness, conscientiousness, extraversion, agreeableness, and neuroticism; see, for example, Staw and Cohen-Charash, 2005). In the light of previous comments, future investigations of this nature should also incorporate varied sources of “external” factors in the workplace/organization, such as: ethical organizational climate and organizational policy, likely to impinge on perceptions of organizational justice and, ultimately, on workers' sense of self, work motivation, and productivity (e.g., Arifin, 2020).

3. We can learn from the lack of significant differences between the three samples employed in the current study. In themselves, each sub-sample is representative of the whole set to a great extent. However, the *total* sample is more representative of the population, such that one may assume the relationships that were found within that composite sample do better resemble reality. As per the central limit theorem, estimating that the larger a sample size in a given set is (i.e., *n* → ∞), the more its distribution approximates a normal distribution (e.g., Rosenblatt, 1956). Therefore, we recommend using as large a sample size as is humanly possible, especially in cross-sectional studies (see also Limitations below).

4. In this investigation, we employed the *moderated-mediation approach*, which we noted was somewhat underused in statistical analysis of these kinds of investigations. In this instance, the lack of the moderating power of LMX might have brought this method into question. Nevertheless, in our opinion, given the possibilities to explain this outcome outlined above, there does not appear to be an objective reason not to replicate the employment of this procedure in further investigations according to the recommendations indicated above.

### Limitations

Further to the discussion above, we now turn to specific limiting factors within the paradigm of this current research.

1. Self-report questionnaires are by nature subjective despite design attempts to overcome personal biases, prejudices, or preconceived notions about what constitutes negative behavior at work. Furthermore, even under conditions of anonymity, individuals might find it difficult to admit to behaviors, such as: theft, sabotage, or disparagement of others—even to themselves. Asking respondents to judge their hostile conduct at work is problematic due to denial processes that operate in the subconscious or because of the threats to one's self-esteem operating when coming to terms with the one's adverse behaviors.

Thus, the CWB questionnaire possibly poses a threat, and respondents are hesitant to report their misdeeds and poor relationships with others at work. This observation is supported by a similar study conducted by Chernyak-Hai and Tziner (2014), which revealed almost identical results for measurements of CWB. The results of such questionnaires are thus questionable. Indeed, in contradistinction to OCB, the effects on CWB as an outcome in our investigation were weak or non-significant.

2. As such, we might suggest that CWB measures obtained by (external) supervisors and co-workers might validate the results obtained by the subjective self-report questionnaires. However, objective these extraneous reports might be they also raise ethical issues concerning colleagues reporting on the “so-called” misdeeds of others at work for whom they may hold biased preferences or prejudices. Indeed, Berry et al. (2012) noted that the inter-rater reliability of “other-reported” measures/scales of CWB is typically low.

3. Additionally, we indicate that the single-sourced and cross-sectional data collected in the investigation was restrictive. Because it does not allow for corroboration of findings over time, the data limits the generalizability of the research.

4. Notably, our research was not directed toward a specific industry, sector, or type of employee, a point in favor of enhancing the external validity of the research. However, that approach also limits the construct validity of the results. These latter comments bring to mind.

Delery and Doty's (1996) observation, noted in the preliminary discussion. Based on contingency theory, they asserted that the optimal way to organize a company depends on the internal and external situation *pervading in that company at any one time*. This axiom raises the more profound question of whether any replication, further in time, can be considered an accurate, valid replication, as external and internal circumstances are continuously subject to change.

## Data Availability Statement

The data set(s) generated and analyzed during the current study are not publicly available due to discretion and anonymity considerations, but are available from the corresponding author on reasonable request.

## Ethics Statement

The current study was correlational, based on a survey, and not a manipulation of subjects. At the beginning of each questionnaire, we explained the general goal of the research, and informed consent was obtained from every participant included in the study. We ensured anonymity and discretion of the results and also ensured that the subjects knew they could drop their participation at any time they chose.

## Author Contributions

All authors listed have made a substantial, direct and intellectual contribution to the work, and approved it for publication.

## Conflict of Interest

The authors declare that the research was conducted in the absence of any commercial or financial relationships that could be construed as a potential conflict of interest.
